# Efficacy of chlorhexidine in alcohol versus aqueous povidone-iodine for preoperative skin antisepsis in preventing surgical site infections: a systematic review and meta-analysis with trial sequential analysis

**DOI:** 10.1186/s12893-026-03958-1

**Published:** 2026-06-15

**Authors:** Liangdong Zhu, Zhenxue Han, Kun Wen, Lan Zhang, Jiefu Tang, Xia Chen

**Affiliations:** 1 The First Hospital of Changsha, Changsha, China; 2 HuaiHua Central Hospital, Huaihua, China; 3 The First Affiliated Hospital of Hunan University of Medicine, Huaihua, China

**Keywords:** Surgical site infection, Chlorhexidine, Povidone-iodine, Meta-analysis

## Abstract

**Background:**

Surgical site infections represent a prevalent postoperative complication, significantly impacting patient safety and increasing the burden on healthcare resources. As a key measure for preventing these infections, preoperative skin antisepsis plays a crucial role. However, current meta-analyses and international guidelines have yet to reach a unified agreement on the optimal type and concentration of antiseptic preparation. This study aims to compare the efficacy of chlorhexidine in alcohol and aqueous povidone-iodine in preventing surgical site infections through systematic review and meta-analysis.

**Methods:**

A systematic search was conducted in PubMed, Embase, Cochrane Library, and Web of Science databases for randomized controlled trials (RCTs) comparing the preoperative skin antisepsis effects of chlorhexidine in alcohol and aqueous povidone-iodine up to May 31, 2025. The primary outcome was the incidence of surgical site infections, while secondary outcomes included adverse events. Subgroup analyses were conducted according to chlorhexidine concentration, wound classification, and surgical category. The meta-analysis was conducted using R software, with a statistical threshold of *P* < 0.05 for significance. To evaluate the robustness of the findings, sensitivity analysis and trial sequential analysis (TSA) were conducted.

**Results:**

The meta-analysis included 17 RCTs involving 13,737 patients. The results revealed that chlorhexidine in alcohol was significantly more effective than aqueous povidone-iodine in preventing surgical site infections (RR = 0.84, 95% CI: 0.73–0.95, *P* < 0.01; I² = 14%). Subgroup analyses further demonstrated that 2.0%-2.5% chlorhexidine in alcohol (RR = 0.83, 95% CI: 0.71–0.97, *P* = 0.02; I² = 32%) was associated with a lower SSI risk than aqueous povidone-iodine. Furthermore, in non-clean surgery (RR = 0.85, 95% CI: 0.75–0.97, *P* = 0.01; I² = 0%) and general surgery (RR = 0.70, 95% CI: 0.53–0.92, *P* = 0.01; I² = 65%), chlorhexidine in alcohol was associated with a significantly lower SSI risk. Sensitivity analyses showed consistent results across individual studies, and TSA suggested that the accumulated evidence has reached the required information size. However, the interpretation of subgroup findings should consider residual heterogeneity in some subgroups and the risk of bias of included trials.

**Conclusion:**

Compared with aqueous povidone-iodine, chlorhexidine in alcohol was associated with a lower risk of surgical site infections, particularly in non-clean surgery and at chlorhexidine concentrations of 2.0%-2.5%.

**Supplementary Information:**

The online version contains supplementary material available at 10.1186/s12893-026-03958-1.

## Introduction

Surgical site infections (SSIs) are among the most prevalent complications following surgery and pose a serious threat to patient safety [1]. The occurrence of SSIs not only extends hospital stays and increases the likelihood of reoperation and readmission but also imposes a significant burden on healthcare resources and economic costs, ultimately negatively affecting the quality and outcomes of surgical procedures [2]. Despite ongoing improvements in surgical techniques and perioperative management in recent years, SSIs remain responsible for 20% of all hospital-acquired infections, with higher prevalence observed among patients in low- and middle-income countries, those undergoing complex surgeries, and those with underlying comorbidities [3]. Therefore, effective prevention of SSIs remains a critical issue in the surgical field that demands urgent attention.

Preoperative skin antisepsis is one of the essential measures for preventing SSIs, aiming to reduce the microbial load on the skin at the surgical incision site, thereby lowering the risk of pathogen contamination during surgery [4]. In clinical practice, several antiseptic preparations are used for preoperative skin antisepsis. Chlorhexidine in alcohol, alcohol-based povidone-iodine, and aqueous povidone-iodine are commonly used depending on the surgical setting and anatomical site [5]. Chlorhexidine in alcohol has a fast onset, broad antimicrobial spectrum, and strong residual antibacterial effects, while aqueous povidone-iodine works by releasing free iodine, which is rapidly bactericidal but has poor persistence. These two differ significantly in their pharmacological mechanisms, modes of use, and antimicrobial spectra, resulting in varying recommendations across different guidelines [5, 6]. Currently, guidelines from the World Health Organization (WHO) and the National Institute for Health and Care Excellence (NICE) advocate for the utilization of chlorhexidine combined with alcohol, whereas the Centers for Disease Control and Prevention (CDC) in the US propose that any antiseptic solution containing alcohol may be employed [7–9]. In addition, existing guidelines provide limited recommendations regarding the optimal concentration of chlorhexidine, despite substantial variation in concentrations used across randomized trials and clinical practice, commonly including 0.5%, 2.0%, 2.5%, and 4.0% formulations. This issue is clinically important because antiseptic concentration may influence antimicrobial efficacy, skin persistence, and tolerability [10, 11].

Although several randomized controlled trials (RCTs) have directly compared chlorhexidine in alcohol with aqueous povidone-iodine for the prevention of SSIs, their findings remain inconsistent [12–14]. However, previous evidence syntheses, including recent meta-analyses by Yang et al. and Wang et al., evaluated broader comparisons across different antiseptic formulations and clinical settings. Importantly, some analyses combined aqueous povidone-iodine with alcohol-based iodine preparations, despite the distinct antimicrobial contribution of the alcohol vehicle itself [15, 16]. Consequently, these broader intervention classifications may have obscured the specific comparative effect of chlorhexidine in alcohol versus aqueous povidone-iodine.

To address this evidence gap, we conducted a systematic review and meta-analysis of RCTs to compare the efficacy and safety of chlorhexidine in alcohol versus aqueous povidone-iodine for preoperative skin antisepsis in adult surgical patients. Prespecified subgroup analyses were performed according to chlorhexidine concentration, wound classification, and surgical category to explore potential modifiers of the treatment effect. In addition, we performed trial sequential analysis (TSA) to determine whether the cumulative evidence was sufficiently robust and conclusive.

## Materials and methods

This systematic review and meta-analysis followed the PRISMA guidelines and was registered in PROSPERO (registration number CRD420251038956) [17].

### Search strategy

Systematic searches were conducted in PubMed, Embase, the Cochrane Library, and Web of Science from database inception to May 31, 2025. The search terms encompassed: “surgical site infection,” “chlorhexidine,” “povidone-iodine,” “randomized controlled trial,” along with other pertinent phrases, integrated using logical combinations of Medical Subject Headings (MeSH) and unrestricted keywords. Additionally, to guarantee the search’s comprehensiveness, manual reviews of reference lists and associated articles were undertaken (Table S1).

### Inclusion criteria and exclusion criteria

The RCTs incorporated in this study need to satisfy the following conditions: (1) the study population consists of adult patients undergoing any type of surgery; (2) the intervention involves preoperative skin antisepsis using different concentrations of chlorhexidine in alcohol; (3) The control group was required to use aqueous povidone-iodine (povidone-iodine without alcohol); (4) Surgical site infection (SSI) was defined as the primary outcome of interest for this meta-analysis. Studies were eligible if they reported extractable SSI data for each intervention group, regardless of whether SSI was prespecified as the primary outcome in the original trial. Secondary outcomes included adverse skin events related to study medication, which were extracted from the included studies as reported. Studies were included if they reported sufficient data on SSI outcomes for quantitative synthesis; (5) the study design is a randomized controlled trial (RCT), fully published, with no language restrictions. Studies were excluded if they were abstracts, reviews, observational studies, duplicates, or had incomplete data or unavailable full texts.

### Data extraction and quality assessment

Two researchers independently (L.Z., Z.H.) reviewed the literature (titles and abstracts) and re-evaluated the full texts based on the inclusion and exclusion criteria to identify eligible studies. In cases of disagreement, a third researcher was consulted to reach the final decision. Data collection encompassed details such as author names, publication years, study sample sizes, counts of SSIs, intervention and control protocols (including drug varieties and dosages), reported adverse events, and definitions of SSIs. SSIs outcomes and their definitions were extracted according to the criteria reported in each original study. Because the included trials varied in surgical procedures and study settings, no post hoc redefinition or reclassification of SSI outcomes was performed in this meta-analysis. When data were incomplete or unclear, corresponding authors were contacted by email for additional information. The final dataset underwent a rigorous accuracy check before being employed for statistical analysis.

The quality of the included RCTs was evaluated using the Cochrane Risk of Bias 2.0 tool. The assessment covered the following aspects: bias due to randomisation, bias due to deviations from intended intervention, bias due to missing data, bias due to outcome measurement, and bias due to selection of reported result. All potential biases were evaluated and classified as “high,” “some concerns,” or “low” risk [18]. Two researchers conducted the assessment independently, with discrepancies resolved through discussion with a third researcher.

### Statistical analysis

Statistical computations were carried out utilizing R software (version 4.4.1) [19]. For binary outcomes, risk ratios (RR) along with their corresponding 95% confidence intervals (CI) were computed, and a random-effects model was employed to derive the overall effect estimate [20]. Heterogeneity of the study was evaluated using I^2^ statistical methods and the χ² test [21, 22]. To bolster the reliability of the findings, TSA was incorporated into the analytical process [23]. TSA effectively controls the risk of Type I errors caused by repeated testing and evaluates whether the current cumulative evidence has reached the required information size (RIS), thereby determining whether the study results are conclusive or require further research. TSA analysis was conducted using TSA software (version 0.9.5.10 Beta), with *α* set to 0.05, *β* set to 0.20 (80% test power), and RIS calculated based on the event rate in the control group, combined effect size, and adjusted heterogeneity. TSA plotted cumulative Z curves to assess whether they crossed traditional significance thresholds, monitoring boundaries, or had reached RIS [24, 25]. Subgroup analyses were performed according to prespecified variables, including chlorhexidine concentration, wound classification, and surgical category. To confirm the stability of the meta-analysis findings, a sensitivity analysis was conducted using the leave-one-out approach, where each study was sequentially excluded to monitor shifts in the pooled effect estimate. This process helped determine if any single study unduly influenced the overall effect size, thereby ensuring the credibility of the results. For studies that included over 10 trials, funnel plots and the Egger test were employed to evaluate potential publication bias [26]. Studies in which neither group reported any SSIs were excluded from the quantitative synthesis.

## Results

### Literature screening and basic characteristics

**Fig. 1:**
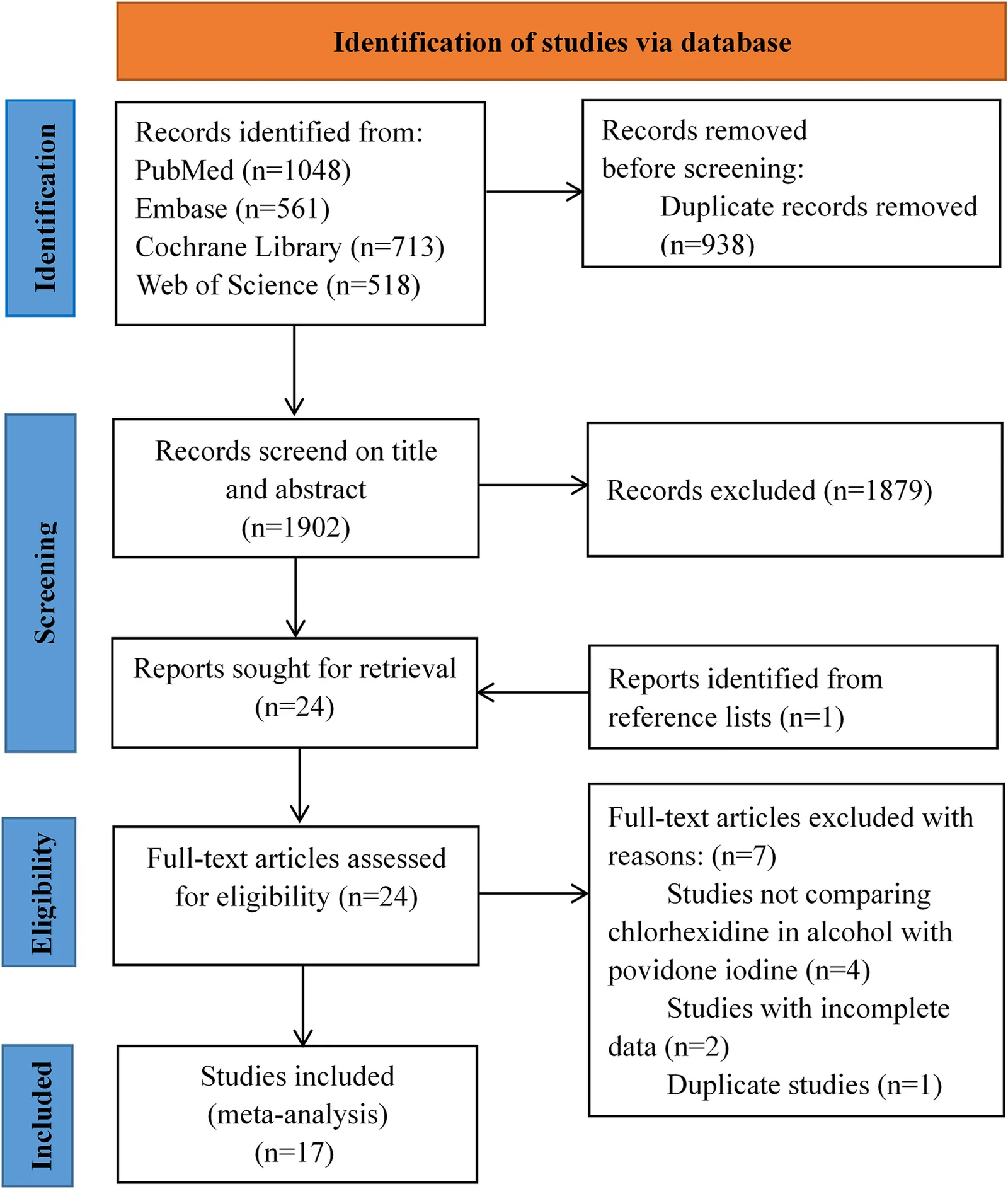
Study selection flow chart

A total of 2,840 records were identified through searches of four electronic databases. After the removal of 938 duplicates, 1,902 records underwent title and abstract screening, of which 1,879 were excluded. One additional potentially eligible record was identified through a manual search of the reference lists of relevant articles. Accordingly, 24 full-text articles were assessed for eligibility. Of these, seven were excluded: four did not compare alcohol-based chlorhexidine with povidone-iodine, two had incomplete data, and one was a duplicate publication. Ultimately, 17 studies were included in the meta-analysis (Fig. 1) [12–14, 27–40].

The combined sample size across these 17 RCTs amounted to 13,737 patients. Among these trials, two compared the effectiveness of 4.0% chlorhexidine in alcohol with aqueous povidone-iodine [31, 34], one compared 2.5% chlorhexidine in alcohol with aqueous povidone-iodine [35], 11 compared 2.0% chlorhexidine in alcohol with aqueous povidone-iodine [13, 14, 27, 29, 30, 32, 33, 36, 37, 39, 40], and the remaining three trials compared the efficacy of 0.5% chlorhexidine in alcohol with aqueous povidone-iodine in preventing SSIs [12, 28, 38]. Given that only one study evaluated 2.5% chlorhexidine in alcohol, the 2.0% and 2.5% concentrations were combined into a single subgroup, defined as the 2.0%-2.5% chlorhexidine in alcohol group, to ensure stability in the analysis. The statistical details of the studies included in this meta-analysis are provided in Table S2.

### Quality assessment

Among the 17 RCTs, five trials were judged to be at low risk of bias [13, 14, 30, 36, 37], ten raised some concerns [12, 27, 28, 31–34, 38–40], and two were judged to be at high risk of bias [29, 35]. The primary contributors to bias involved flaws in randomization processes and deviations from the planned interventions (Figure S1). On the whole, the quality of the studies included spanned from moderate to high (Figure S2).

### Primary outcome analysis results

**Fig. 2:**
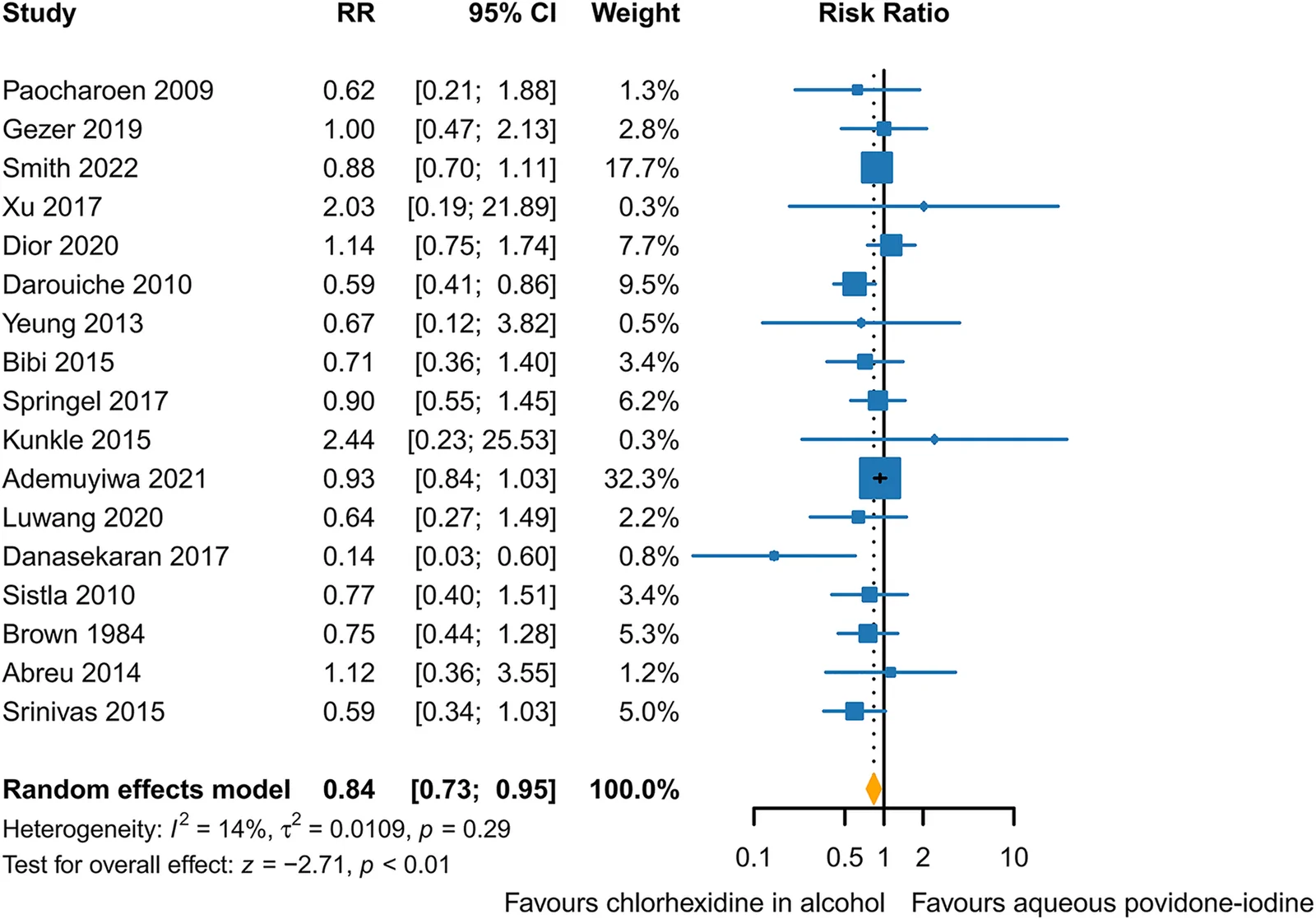
Forest plot of chlorhexidine in alcohol vs. aqueous povidone-iodine for surgical site infection prevention

**Fig. 3:**
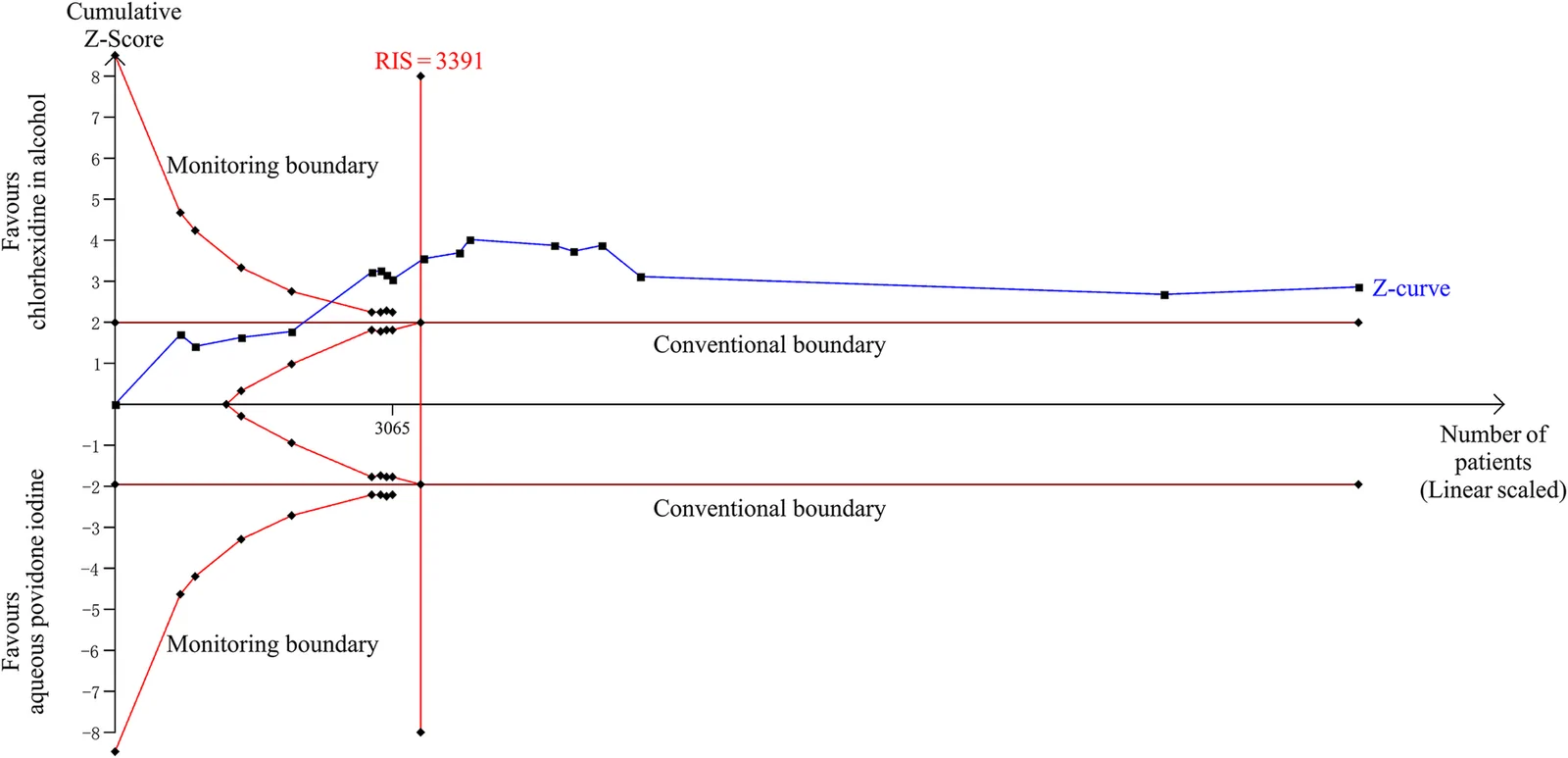
Trial sequential analysis. The cumulative Z-curve crossed both the conventional boundary and the monitoring boundary, and exceeded the required information size, indicating that sufficient evidence has been obtained and conclusive results have been reached. The likelihood of subsequent research altering the overall conclusions is minimal. A diversity-adjusted required information size of 3391 patients was calculated based on a 5% type I error (two-sided) and a 20% type II error (80% power). RIS, required information size

The observed baseline risk of SSIs in the aqueous povidone-iodine group was 149.0 per 1,000 patients. Based on the pooled effect estimate, the corresponding risk in the chlorhexidine in alcohol group was estimated to be 125.2 per 1,000 patients. This translated into an absolute risk reduction of 23.8 SSIs per 1,000 patients (95% CI, 7.5–40.2 per 1,000) and a number needed to treat of 42 (95% CI, 25–135). The impact of chlorhexidine in alcohol versus aqueous povidone-iodine on preventing SSIs as the primary outcome was evaluated (Fig. 2). The findings revealed that chlorhexidine in alcohol significantly reduced the occurrence rate of SSIs in comparison to aqueous povidone-iodine (RR = 0.84, 95% CI: 0.73–0.95, *P* < 0.01), with minimal heterogeneity observed among the studies (I² = 14%, τ² = 0.0109, *P* = 0.29). Sensitivity analysis using a leave-one-out approach showed that sequential exclusion of individual studies did not materially change the pooled effect estimates, indicating that the overall findings were not driven by any single study (Figure S3). Subsequently, TSA was performed to evaluate the primary outcome effect. The TSA analysis indicated that the cumulative Z-curve exceeded the conventional significance level and reached the RIS (Fig. 3). This indicates that the current sample size is sufficient to provide adequate statistical power, with the results being statistically significant and random errors effectively controlled. Therefore, TSA suggests that the current cumulative evidence is sufficient to demonstrate a statistically significant association. Nevertheless, given the heterogeneity observed in certain subgroups and the presence of studies with some concerns or high risk of bias, future high-quality trials may still refine the magnitude of effect in specific clinical contexts. The comparison-adjusted funnel plot appeared symmetrical (Figure S4), and the Egger test indicated no statistically significant difference (*P* = 0.13), indicating a low risk of publication bias.

### Subgroup analysis results

**Fig. 4:**
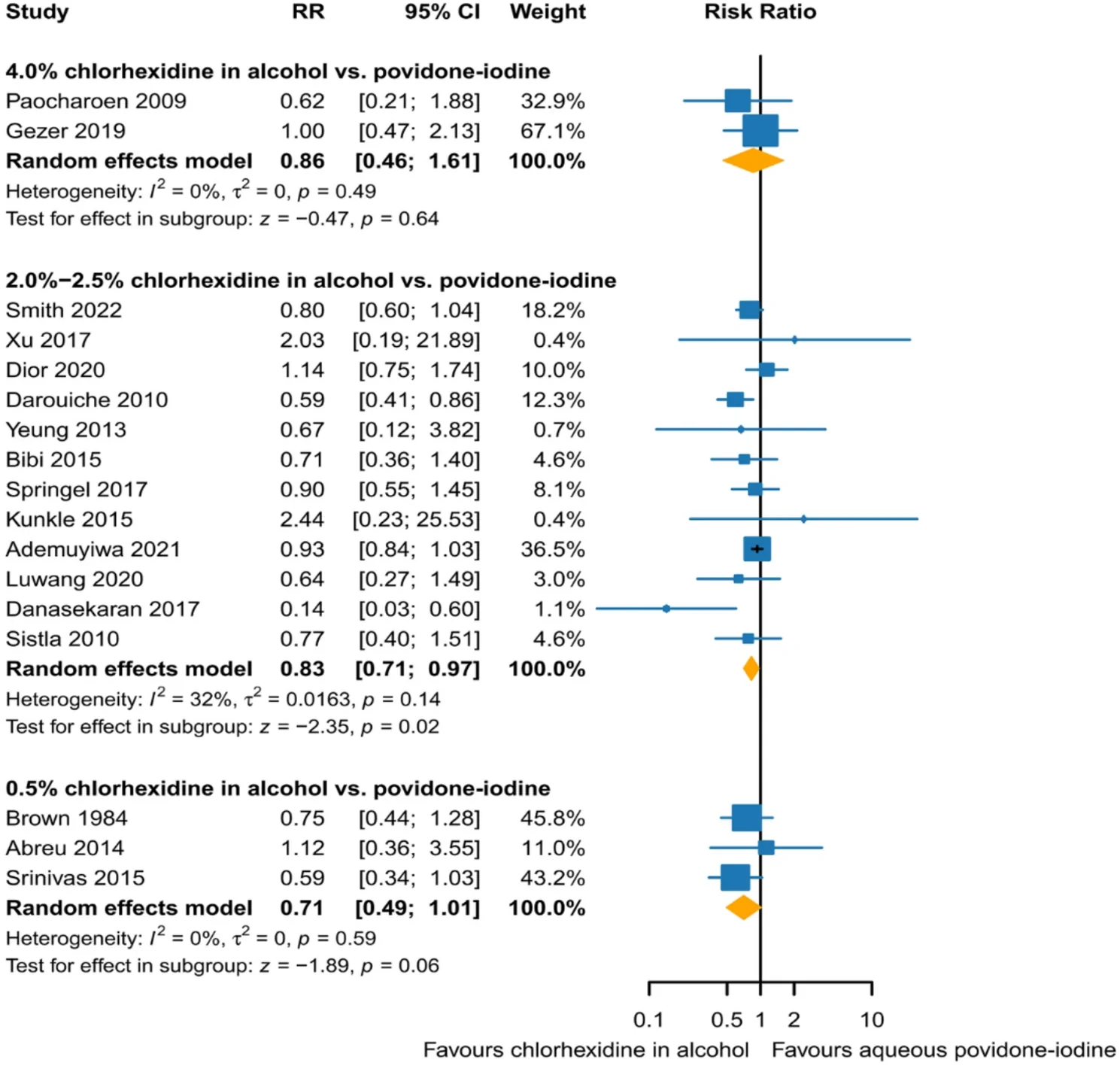
Forest plot for different concentrations

The efficacy of different concentrations of chlorhexidine in alcohol and aqueous povidone-iodine for preventing SSIs was assessed (Fig. 4). The analysis showed that 2.0%-2.5% chlorhexidine in alcohol was associated with a significantly lower SSI risk than aqueous povidone-iodine (RR = 0.83, 95% CI: 0.71–0.97, *P* = 0.02), with low heterogeneity (I² = 32%, τ² = 0.0163, *P* = 0.14). The 0.5% chlorhexidine in alcohol group showed a trend toward a reduced risk of SSIs; however, this difference did not reach statistical significance (RR = 0.71, 95% CI: 0.49–1.01, *P* = 0.06), with low heterogeneity (I² = 0%, τ² = 0, *P* = 0.59). Similarly, no statistical difference was observed between the 4.0% chlorhexidine in alcohol group and aqueous povidone-iodine group (RR = 0.86, 95% CI: 0.46–1.61, *P* > 0.05), with low heterogeneity (I² = 0%, τ² = 0, *P* = 0.49).

**Fig. 5:**
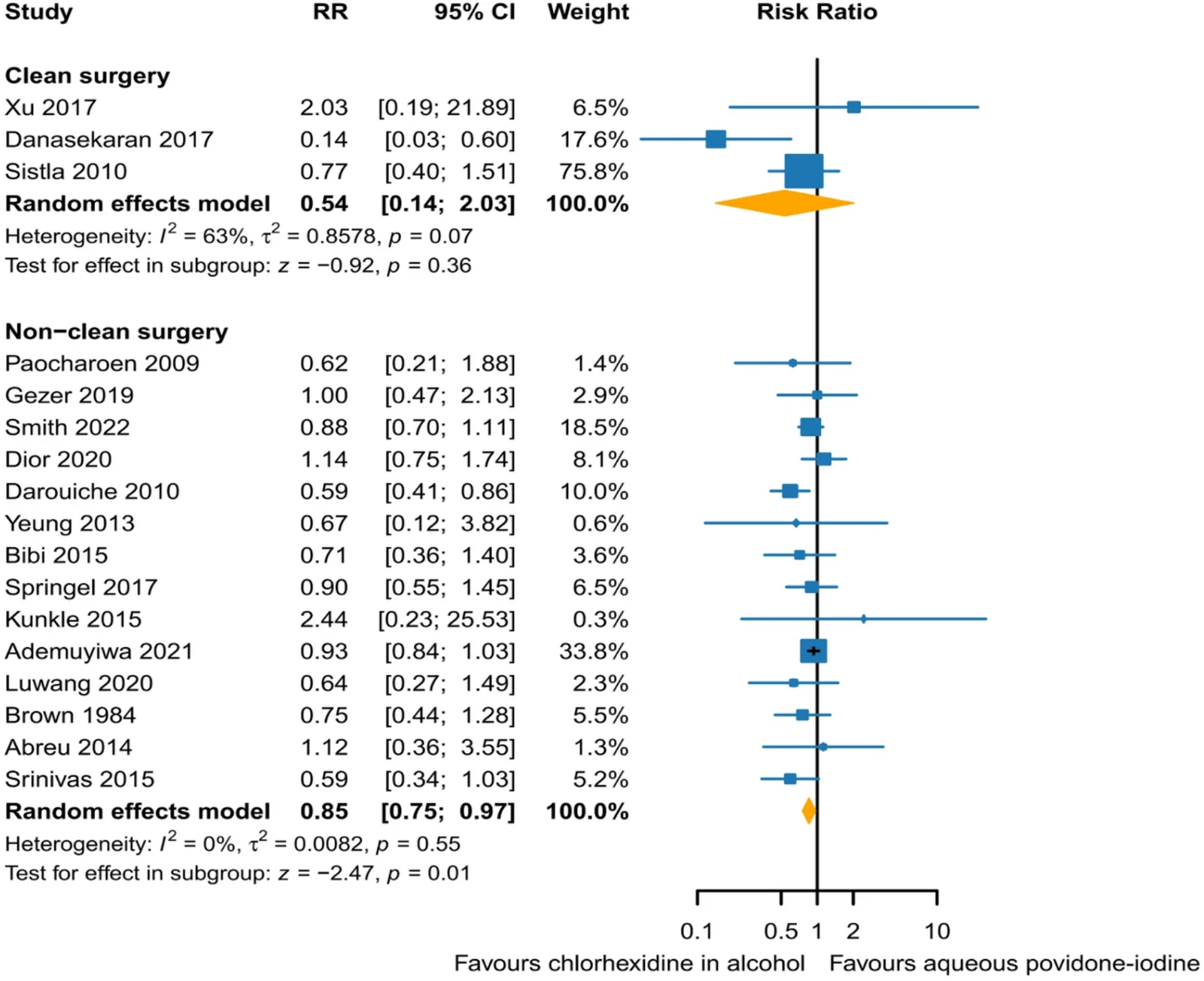
Forest plot by wound classification

The efficacy of chlorhexidine in alcohol and aqueous povidone-iodine was assessed according to wound classification (Fig. 5). The results showed no statistically significant difference in SSI incidence between the chlorhexidine in alcohol and aqueous povidone-iodine groups in clean surgeries; however, in non-clean surgery, chlorhexidine in alcohol showed significantly better preventive effects (RR = 0.85, 95% CI: 0.75–0.97, *P* = 0.01), with low heterogeneity (I² = 0%, τ² = 0.0082, *P* = 0.55).

**Fig. 6:**
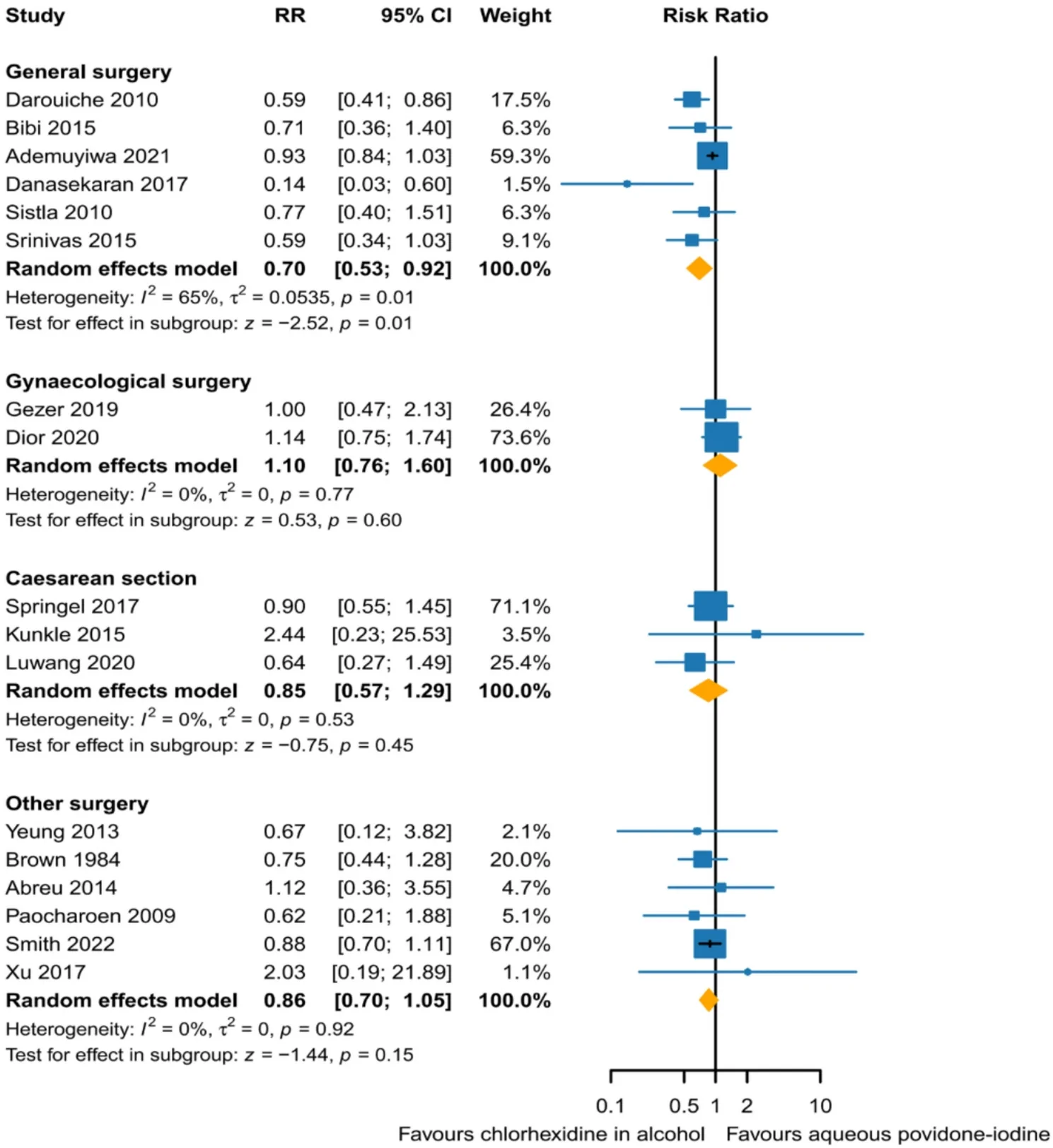
Forest plot by surgical category

The efficacy of chlorhexidine in alcohol and aqueous povidone-iodine was assessed according to surgical category (Fig. 6). The results showed that in general surgery, the SSIs incidence in the chlorhexidine in alcohol group was significantly lower than in the aqueous povidone-iodine group (RR = 0.70, 95% CI: 0.53–0.92, *P* = 0.01), with high heterogeneity between studies (I² = 65%, τ² = 0.0535, *P* = 0.01). However, no significant differences were observed between the two groups in gynecological surgeries, cesarean sections, and other surgery.

### Adverse events analysis

Among the 17 RCTs included in the analysis, 8 studies explicitly reported skin adverse events associated with preoperative skin antiseptics. 5 studies reported no adverse events, while the remaining three studies recorded 10 minor adverse reactions, mainly including erythema, itching, dermatitis, skin irritation, or mild allergic symptoms. All adverse events were temporary, reversible reactions, and did not lead to serious complications. No statistically significant differences in reported adverse events were observed between groups, although safety reporting was limited and inconsistent across trials.

## Discussion

In this systematic review and meta-analysis of 17 RCTs involving 13,737 adult surgical patients, chlorhexidine in alcohol was associated with a significantly lower risk of SSIs than aqueous povidone-iodine. Although statistically significant, the overall effect was modest, and between-study heterogeneity was low. When the pooled RR was applied to the observed baseline risk in the aqueous povidone-iodine group, the estimated absolute risk reduction was 23.8 per 1,000 patients (95% CI, 7.5–40.2 per 1,000), corresponding to a number needed to treat of 42 (95% CI, 25–135). TSA showed that the cumulative evidence reached the RIS for the primary outcome, indicating that this finding is unlikely to be attributable solely to random error. Taken together, these results suggest that chlorhexidine in alcohol is more effective than aqueous povidone-iodine for preoperative skin antisepsis, although the magnitude of the absolute benefit remains modest.

Previous evidence syntheses have evaluated the comparative effectiveness of antiseptic formulations and concentrations using broader intervention classifications [15, 16, 41, 42]. For example, Yang et al. analyzed 14 RCTs comparing 2.0%-2.5% chlorhexidine in alcohol with aqueous or alcohol-based iodine solutions and reported a reduction in SSI risk, although substantial heterogeneity was observed (RR = 0.30, 95% CI: 0.20–0.46; I² = 95%) [15]. More recently, Wang et al. assessed chlorhexidine and povidone-iodine across different formulations and clinical settings in a meta-analysis of 26 RCTs [16]. By contrast, our analysis was restricted to direct comparisons between chlorhexidine in alcohol and aqueous povidone-iodine, thereby avoiding formulation heterogeneity attributable to the inclusion of alcohol-based iodine preparations. In addition, we evaluated multiple chlorhexidine concentrations and incorporated absolute effect estimates and trial sequential analysis. Thus, our findings provide a more clinically specific interpretation of the available evidence: chlorhexidine in alcohol was associated with a lower risk of SSI than aqueous povidone-iodine, whereas its comparative effectiveness against alcohol-based povidone-iodine remains uncertain.

The concentration subgroup analysis offers additional clinically relevant insights, although these findings should be interpreted cautiously. The clearest benefit was observed for 2.0%-2.5% chlorhexidine in alcohol, whereas 0.5% chlorhexidine showed only a non-significant trend and 4.0% chlorhexidine did not demonstrate a clear additional advantage. These findings do not support a simple dose-response relationship whereby higher chlorhexidine concentrations necessarily lead to greater SSI prevention. The clinical effect of chlorhexidine in alcohol is likely influenced not only by chlorhexidine concentration but also by alcohol type and concentration, application technique, drying time, residual activity, anatomical site, and baseline contamination risk [43–45]. Therefore, the current evidence suggests that 2.0%-2.5% chlorhexidine in alcohol has the strongest supporting evidence within this comparison, but it does not establish that 4.0% preparations are clinically superior.

The subgroup analysis by wound classification also has important implications. Chlorhexidine in alcohol showed a clearer benefit in non-clean surgery, whereas no statistically significant difference was observed in clean surgery. This pattern is biologically plausible. In clean surgery, the baseline risk of SSI is relatively low, and the incremental benefit of a more active antiseptic may be small and difficult to detect. In contrast, clean-contaminated and other non-clean procedures may involve a higher microbial burden and greater baseline infection risk, making the rapid bactericidal effect of alcohol and the residual antimicrobial activity of chlorhexidine more likely to translate into measurable clinical benefit [46, 47]. These findings suggest that antiseptic choice may be particularly relevant in procedures with higher baseline infection risk. However, the absence of a statistically significant difference in clean surgery should not be interpreted as evidence of equivalence, because event rates were lower and statistical power may have been limited.

The surgical category subgroup analysis showed a significant benefit in general surgery, but this finding should be interpreted cautiously because heterogeneity was substantial in this subgroup. General surgery includes a wide range of procedures with different incision sites, contamination levels, perioperative antibiotic strategies, operative durations, patient risk profiles, and postoperative surveillance practices. Therefore, the observed benefit may reflect both the pharmacological effect of the antiseptic agent and differences in baseline SSI risk across procedure types. No statistically significant difference was observed in gynecological surgery, cesarean section, or other surgical categories. However, these findings should not be interpreted as evidence that chlorhexidine in alcohol is ineffective in these settings, because several subgroup analyses included limited numbers of trials and events.

An important strength of this study is the use of TSA. Conventional meta-analysis may yield statistically significant findings before the accumulated sample size is sufficient, particularly when new trials are repeatedly added over time [23]. In the present analysis, the cumulative Z-curve crossed the conventional significance boundary and reached the RIS, supporting the statistical robustness of the overall comparison. However, TSA does not resolve all uncertainty. It addresses random error and information size, but it cannot correct for clinical heterogeneity, differences in SSI definitions, variation in perioperative care, incomplete safety reporting, or risk of bias within the included trials. Therefore, the overall result can be considered statistically robust, whereas subgroup findings should remain exploratory.

The comparison with a recent RCT also requires careful interpretation. Widmer et al. reported that alcohol-based povidone-iodine was noninferior to chlorhexidine in alcohol in patients undergoing cardiac or abdominal surgery, with similar SSI rates between groups [48]. This finding is not inconsistent with the present meta-analysis, because our comparator was aqueous povidone-iodine rather than alcohol-based povidone-iodine. Rather, it reinforces the view that the alcohol component may contribute substantially to antiseptic efficacy. Therefore, our findings should not be extrapolated to conclude that chlorhexidine in alcohol is superior to all iodine-based preparations. The more precise conclusion is that chlorhexidine in alcohol appears superior to aqueous povidone-iodine, while its comparative effectiveness against alcohol-based povidone-iodine remains uncertain.

Safety is another important consideration. The adverse events reported in the included trials were uncommon, mild, and reversible, including erythema, pruritus, dermatitis, skin irritation, and mild allergic symptoms. No significant difference in adverse events was observed between chlorhexidine in alcohol and aqueous povidone-iodine. However, safety reporting was incomplete across studies, and the absence of a significant difference should not be interpreted as definitive equivalence. Chlorhexidine has been associated with rare but potentially serious hypersensitivity reactions, while alcohol-based preparations require adequate drying time to reduce fire-related risk in the operating room [49, 50]. Future trials should use standardized adverse-event definitions and systematically report skin reactions, allergic events, mucosal exposure, chemical irritation, alcohol-related complications, and safety outcomes related to drying time and application technique.

This study has several limitations. First, the comparator was intentionally restricted to aqueous povidone-iodine. This allowed a more focused clinical comparison; however, it also limits the generalizability of our findings to alcohol-based iodine preparations. Additional direct evidence across diverse surgical populations is still needed to clarify the comparative effectiveness of chlorhexidine in alcohol and alcohol-based povidone-iodine. Second, the included trials differed in SSI definitions, follow-up duration, surgical populations, wound classification, perioperative antibiotic protocols, antiseptic application methods, and surveillance strategies. Third, although the overall heterogeneity was low, some subgroups, especially general surgery, showed substantial heterogeneity. Fourth, several subgroup analyses included relatively few trials and events, limiting statistical power and increasing the risk of unstable estimates. Fifth, some included trials had unclear or high risk of bias, which may influence the reliability of the pooled estimates. Finally, cost-effectiveness was not evaluated, although economic considerations are important for guideline implementation, particularly in resource-limited settings.

## Conclusion

Chlorhexidine in alcohol was associated with a statistically significant reduction in SSI risk compared with aqueous povidone-iodine in adult surgical patients, and this finding was supported by TSA. The benefit appeared most consistent for 2.0%-2.5% chlorhexidine in alcohol and in non-clean surgery, although subgroup findings should be interpreted cautiously. These results support the preferential consideration of chlorhexidine in alcohol over aqueous povidone-iodine when clinically appropriate, but should not be extrapolated to alcohol-based povidone-iodine. Future studies should clarify the comparative effectiveness of alcohol-based antiseptic formulations and improve the standardization of SSI, adverse-event, and cost-effectiveness reporting.

## Supplementary Information

Supplementary Material 1.

## Data Availability

The data generated and analyzed during this study are available in the article and its Supplementary Materials.
