# Supplementary material for: Efficacy of chlorhexidine in alcohol versus aqueous povidone-iodine for preoperative skin antisepsis in preventing surgical site infections: a systematic review and meta-analysis with trial sequential analysis
Source: BMC Surg. 2026 Jun 15;26:562. doi: 10.1186/s12893-026-03958-1 (PMC13495349; doi:10.1186/s12893-026-03958-1)
Supplement: Supplementary file 1 [file 12893_2026_3958_MOESM1_ESM.docx]

**Supplementary Materials**

Table S1. Search strategy.

Table S2. Study characteristics.

Figure S1. Risk of bias graph.

Figure S2. Risk of bias summary.

Figure S3. The forest plot of sensitivity analysis.

Figure S4. Comparison-adjusted funnel plot.

Table S1. Search strategy

| **Database** | **Search strategy** |
| --- | --- |
| Pubmed | ("Surgical Wound Infection"[Mesh] OR Surgical Wound Infection*[Title/Abstract] OR Surgical Site Infection*[Title/Abstract] OR SSI[Title/Abstract] OR preoperative care*[Title/Abstract]) AND ("Chlorhexidine"[Mesh] OR Chlorhexidine*[Title/Abstract] OR "Povidone-Iodine"[Mesh] OR povidone*[Title/Abstract] OR "Iodine"[Mesh] OR Iodine*[Title/Abstract] OR "Antisepsis"[Mesh] OR antisepsis[Title/Abstract]) AND ("randomized controlled trials"[All Fields] OR "RCT"[All Fields] OR "clinical"[All Fields] OR "trial"[All Fields] OR "trials"[All Fields]) |
| Embase | ('surgical infection'/exp OR 'surgical infection' OR 'surgical infection':ti,ab,kw OR 'surgical wound infection':ti,ab,kw OR ssi:ti,ab,kw) AND ('chlorhexidine'/exp OR 'chlorhexidine' OR 'chlorhexidine':ti,ab,kw OR 'povidone iodine':ti,ab,kw OR 'povidone':ti,ab,kw OR 'iodine':ti,ab,kw OR 'antisepsis':ti,ab,kw) AND ('randomized controlled trial (topic)'/exp OR 'randomized controlled trial':ti,ab,kw OR 'clinical trial':ti,ab,kw OR trial:ti,ab,kw) |
| Cochrane library | ((Surgical Wound Infection*):ti,ab,kw OR (Wound Infection*):ti,ab,kw OR (Surgical Site Infection*):ti,ab,kw OR (SSI):ti,ab,kw) AND (("chlorhexidine"):ti,ab,kw OR ("chlorhexidine gluconate"):ti,ab,kw OR ("povidone-iodine"):ti,ab,kw OR ("povidone"):ti,ab,kw OR ("antisepsis"):ti,ab,kw) AND ((randomized controlled trials):ti,ab,kw OR (trials):ti,ab,kw) |
| Web of Science | (Surgical Site Infection (Topic) or Surgical Wound Infection (Topic) or SSI (Topic)) AND (Chlorhexidine (Topic) or Chlorhexidine gluconate (Topic) or Povidone Iodine (Topic) or Iodine (Topic) or Povidone (Topic) or antisepsis (Topic)) AND (Randomized controlled trials (Topic) or trials (Topic) |

Abbreviations: MeSH terms: Medical Subject Headings; exp: explosion in Emtree searching of selected subject terms and related subjects; ti, ab, kw: title, abstract, keyword fields.

| **Study** | **Total sample size*** | **SSI*** | **Treatment 1** | **Treatment 2** | **Surgical category** | **Adverse events** | **Wound classification** | **SSI definition** |
| --- | --- | --- | --- | --- | --- | --- | --- | --- |
| Paocharoen 2009 | 250/250 | 5/8 | 4.0% CHG in 70% IPA | 10% PI | Not reported | 2 patients with skin irritation | 1，2，3 | A# |
| Gezer 2019 | 110/110 | 12/12 | 4.0% CHG with alcohol | 10% PI | Gynaecological surgery | No reports | 1，2 | CDC |
| Smith 2022 | 703/1062 | 70/133 | 2.0% CHG in 70% IPA | 10% PI | mixed surgery | No events | 1，2，3，4 | CDC |
| Xu 2017 | 79/80 | 2/1 | 2.0% CHG in 70% IPA | 10% PI | Orthopaedic surgery | No reports | 1 | B# |
| Dior 2020 | 210/214 | 38/34 | 2.0% CHG in 70% ethanol | 10% PI | Gynaecological surgery | No reports | 1，2 | CDC |
| Darouiche 2010 | 431/466 | 39/71 | 2.0% CHG in 70% IPA | 10% PI | General surgery | 6 patients with pruritus, erythema, or both | 2 | CDC |
| Yeung 2013 | 50/50 | 2/3 | 2.0% CHG in 70% IPA | 7.5% iodine scrub + 10% PI paint | Urological implant surgery | No events | 2 | No definition |
| Bibi 2015 | 168/220 | 12/22 | 2.0% CHG in 70% IPA | 10% PI | General surgery | 2 patients with itching and rashes | 1，2 | CDC |
| Springel 2017 | 461/471 | 29/33 | 2.0% CHG in 70% IPA | 0.75% AI scrub + 10% PI paint | Caesarean section | No events | 1，2 | CDC |
| Kunkle 2015 | 27/33 | 2/1 | 2.0% CHG in 70% IPA | 10% PI | Caesarean section | No reports | 1，2 | C# |
| Ademuyiwa 2021 | 2892/2896 | 561/602 | 2.0% CHG in 70% IPA | 10% PI | General surgery | No events | 2，3，4 | CDC |
| Luwang 2020 | 153/158 | 8/13 | 2.0% CHG in 70% IPA | 10% PI | Caesarean section | No reports | 1，2 | D# |
| Danasekaran 2017 | 60/60 | 2/14 | 2.0% CHG in 70% IPA | 10% PI | General surgery | No reports | 1 | E# |
| Sistla 2010 | 271/285 | 14/19 | 2.5% CHG in 70% ethanol | 10% PI | General surgery | No reports | 1 | CDC |
| Brown 1984 | 378/359 | 23/29 | 0.5% CHG in 70% IPA | 0.75% AI | mixed surgery | No reports | 1，2，3 | F# |
| Abreu 2014 | 32/24 | 6/4 | 0.5% CHG in alcohol | 0.5% PI | Urological surgery | No reports | 1，2 | CDC |
| Srinivas 2015 | 163/188 | 17/33 | 0.5% CHG in 70% IPA | 5% PI | General surgery | No events | 2 | CDC |

Table S2. Study characteristics

Abbreviations: SSI: Surgical site infection; CHG: Chlorhexidine Gluconate; IPA: isopropyl alcohol; PI: povidone iodine; CDC: The U.S. Centers for Disease Control and Prevention; AI: available iodine. Wound classification: 1, clean; 2, clean-contaminated; 3, contaminated; 4, dirty. * Values are presented as chlorhexidine in alcohol group first, followed by the aqueous povidone-iodine group.

A#: a surgical wound drained purulent material or if the surgeon judges it to be infected and opens it (incisional), within 1 month of surgery. B#: need for antibiotics or surgical intervention, within 6 weeks of surgery. C#: presence of purulent drainage, cellulitis, or the need for incision and drainage, or treatment with antibiotics for a clinical diagnosis of infection, within two weeks of surgery. D#: purulent discharge from the incision site, wound dehiscence, localized pain or tenderness, localized swelling, and erythema or heat within 30 days following caesarean section. E#: for example; purulent/serous discharge from the wound, redness of the surrounding area, pain associated with discharge, increased local temperature, within 10 days of surgery. F#: a minor wound infection was defined as an infected wound with superficial separation (less than 1 centimeter) involving less than one-third of the incision or induration of the wound edge believed by the surgeon to be secondary to infection; a major wound infection was defined. as an infected wound with separation of the wound edges greater than one-third of the length of the incision or frank wound infection with evidence of purulent exudate or abscess.

Figure S1. Risk of bias graph.

Figure S2. Risk of bias summary.

Figure S3. The forest plot of sensitivity analysis.

Figure S4. Comparison-adjusted funnel plot.
